# Engineered β-Lactoglobulin Produced in *E. coli*: Purification, Biophysical and Structural Characterisation

**DOI:** 10.1007/s12033-016-9960-z

**Published:** 2016-07-05

**Authors:** Joanna I. Loch, Piotr Bonarek, Magdalena Tworzydło, Agnieszka Polit, Barbara Hawro, Aneta Łach, Eryk Ludwin, Krzysztof Lewiński

**Affiliations:** 1 Biocrystallography Group, Department of Crystal Chemistry and Crystal Physics, Faculty of Chemistry, Jagiellonian University, Ingardena 3, 30-060 Kraków, Poland; 2 Department of Physical Biochemistry, Faculty of Biochemistry, Biophysics and Biotechnology, Jagiellonian University, Gronostajowa 7, 30-387 Kraków, Poland

**Keywords:** β-Lactoglobulin, *Escherichia coli*, Protein engineering, Protein stability, Ligand binding, N-terminal methionine

## Abstract

Functional recombinant bovine β-lactoglobulin has been produced by expression in *E. coli* using an engineered protein gene and purified to homogeneity by applying a new protocol. Mutations L1A/I2S introduced into the protein sequence greatly facilitate in vivo cleavage of the N-terminal methionine, allowing correctly folded and soluble protein suitable for biochemical, biophysical and structural studies to be obtained. The use of gel filtration on Sephadex G75 at the last purification step enables protein without endogenous ligand to be obtained. The physicochemical properties of recombinant β-lactoglobulin such as CD spectra, ligand binding (*n*, *K*_a_, Δ*H*, *T*Δ*S*, Δ*G*), chemical and thermal stability (Δ*G*_D_, *C*_mid_) and crystal structure confirmed that the protein obtained is almost identical to the natural one. The substitutions of N-terminal residues did not influence the binding properties of the recombinant protein so that the lactoglobulin produced and purified according to our protocol is a good candidate for further engineering and potential use in pharmacology and medicine.

**Electronic supplementary material:**

The online version of this article (doi:10.1007/s12033-016-9960-z) contains supplementary material, which is available to authorized users.

## Introduction

Bovine β-lactoglobulin (BlgB) is a small protein (18.3 kDa) belonging to the lipocalin family. Due to BlgB ability to bind different classes of bioactive compounds, its application as a nutrients nanotransporter or drug carrier is currently an object of intensive studies [1–5]. Most of them are performed with commercially available natural lactoglobulin, but the use of recombinant protein seems to be a better alternative. The expression of correctly folded BlgB was successful for an extended period but only in eukaryotic systems: insect [6], yeast (*P. pastoris* [6], *S. cerevisiae* [8]) and transgenic mice [9]. More recent studies showed that BlgB can be produced and secreted by *Lactobacillus casei* [10] while attempts to express β-lactoglobulin in *E. coli* have continued to fail due to incorrect protein folding. The expression systems used for lactoglobulin production were reviewed in details by Ariyaratne et al. [11].

The strategy for successful expression of β-lactoglobulin in *E. coli* was discovered by Ponniah et al. [12], who produced soluble and correctly folded BlgB in *Origami B* cells. Folding of recombinant protein in the bacteria cytoplasm was possible due to its co-expression with DsbC (*E. coli* cytoplasmic disulphide bond isomerase), which facilitates disulphide bridges formation in the reducing environment of bacterial cytoplasm.

Potential utilisation in medicine of small proteins belonging to the lipocalin family has recently intensified their studies [13]. Such research has been aimed at producing engineered proteins that can specifically recognise receptors or bind desired low molecular weight ligands [14]. An increased affinity to selected molecular targets is achieved by introducing mutations into the binding site region. In modified lipocalins, substituted residues interact specifically with ligands and change the binding pocket plasticity [15].

The process of protein engineering requires an expression system which allows obtaining high yields of pure and biologically active protein molecules. Engineered lipocalins, called *Anticalins*, are usually produced in prokaryotic expression systems; however, due to the presence of disulphide bonds in their structure, during expression they are directed to the periplasmic space (e.g. by N-terminal *OmpA* signal peptide), in which efficient S–S bridge formation occurs. Anticalins also may have affinity tag at the C-terminus to facilitate protein purification [16], but it often needs to be removed in vitro before structural and other studies. This experimental strategy, however, generates additional steps in the protein purification protocol.

As other proteins from lipocalin family, BlgB can be re-engineered to gain specificity of binding selected bioactive ligands, and this requires heterologous protein production in the appropriate expression system. Such system should be not expensive and enable to produce the correctly folded protein in high amounts needed for medical and structural studies. In this context, strategy for BlgB production in *E. coli* with co-expression of the DsbC isomerase [12] seems to be especially valuable. We have repeated this procedure (protocol #1), but we have found that obtained BlgB has physicochemical properties different than natural lactoglobulin isolated from bovine milk. Detailed studies, presented below, revealed the presence of uncleaved N-terminal methionine and endogenous fatty acid tightly bound in the binding pocket which significantly influenced properties of the recombinant protein and affected binding of pharmaceutical agents.

Here, we present a modification of expression and purification protocol (protocol #2) which allows producing in *Origami B* (*DE3*) cells fully functional BlgB. To compare properties of recombinant lactoglobulin produced using different protocols, we used a range of techniques. Secondary and tertiary structure in solution was inspected by CD spectra. Thermal and chemical denaturation monitored by CD spectra changes was used to determine protein stability. The thermodynamic parameters of model ligand binding have been measured using isothermal titration calorimetry. Origins of different properties observed for proteins produced with the use of different protocols were explained on the basis of MS and crystal structures.

## Materials and Methods

### Materials

All chemicals used for protein purification and crystallization, e.g. phosphate and Tris–HCl buffer ingredients, salts: NaCl, (NH_4_)_2_SO_4_, HCl, tri-sodium citrate in analytical grade, were purchased from *IDALIA* (*Poland*) or *Avantor Performance Materials Poland S.A*.

Antibiotics (ampicillin sodium salt, kanamycin monosulphate tetracycline chloride) and LB broth Miller (*BioShop*) were purchased from *LabEmpire*. Agar and IPTG were purchased from *A&A Biotechnology* (*Poland*). Natural (milk) β-lactoglobulin isoform B used as a reference in spectroscopic and calorimetric studies was purchased from *Sigma*-*Aldrich*.

### Construction of Expression Vector and Mutagenesis

Genes coding bovine β-lactoglobulin isoform B (*BlgB,* UNP: P02754) and bacterial disulphide bond isomerase DsbC (*DsbC,* UNP: P0AEG6) were chemically synthesised and optimised for the expression in *E. coli* by GeneArt (Germany). In both cases, the short sequences responsible for the extracellular localisation of proteins were omitted. The expression vector was generated according to Ponniah et al. [12]: *DsbC* was cloned into MCS I while *BlgB* was introduced into MCS II of the pETDuet-1 vector (*Invitrogen*) using *NcoI/HindIII* and *NdeI/KpnI* set of endonucleases. The use of the *NcoI* restriction site eliminated the presence of His-tag at the N-end of DsbC and duplicated TAA codon at the end of *BlgB* preventing the formation of S-tag at the C-end of rBlgB. Before transformation, the modified vector pETDuet-1/*DsbC*/*BlgB* was sequenced to prove that no frameshift or mutation took place during the cloning procedure.

Initial amino acids in β-lactoglobulin were modified by *QuikChange* method. A pair of complementary mutagenic primers (FOR:5′GGAGATATACATATGGCCTCAGT TACCCAGACCATG3′ and REV:5′CATGGTCTGGGTAACTGAGGCCATATGTATATCTCC3′) was applied to replace codons for Leu1 and Ile2 with Ala and Ser during PCR. pETDuet-1/*DsbC*/*BlgB* vector served as a matrix for this reaction. In the next step, the methylated matrix was digested with *DpnI* restriction enzyme and newly synthesised DNA (pETDuet-1/*DsbC*/L1A/I2S-*BlgB*) was used to transform *E. coli* DH5α competent cells. The presence of the desired mutation in obtained colonies was confirmed by sequencing.

### Expression

Competent *E. coli Origami B* (*DE3*) (*Novagen*) cells were transformed with pETDuet-1/*DsbC*/*BlgB* or pETDuet-1/*DsbC*/L1A/I2S-*BlgB* vector and grown overnight in LB–agarose plates containing 100 μg/ml ampicillin, 15 μg/ml kanamycin and 12.5 μg/ml tetracycline. Single colonies were transferred to 5 ml of LB medium containing the same antibiotics and grown overnight at 37 °C. Next day, liquid culture was increased to 50 ml and incubated overnight at 37 °C. Fifty ml of overnight culture was added to 1.2 L of LB media supplemented with the same antibiotics, and cultures were incubated for 3–6 h at 37 °C applying constant stirring 100 rpm, until OD_600_ reached 0.5–0.7 (pETDuet-1/*DsbC*/*BlgB*) or 1.0–1.2 (pETDuet-1/*DsbC*/L1A/I2S-*BlgB* vector). When OD_600_ reached desired value, protein expression was induced by adding IPTG to its final concentration 500 μM. After induction, the temperature was decreased to 25 °C and cultures were incubated for 3–4 h (pETDuet-1/*DsbC*/*BlgB* vector) or overnight (pETDuet-1/*DsbC*/L1A/I2S-*BlgB* vector), with constant stirring 200 rpm. After this time, cells were harvested by centrifugation at 5000 rpm and frozen at −80 °C.

### Purification Protocol #1

**Fig. 1:**
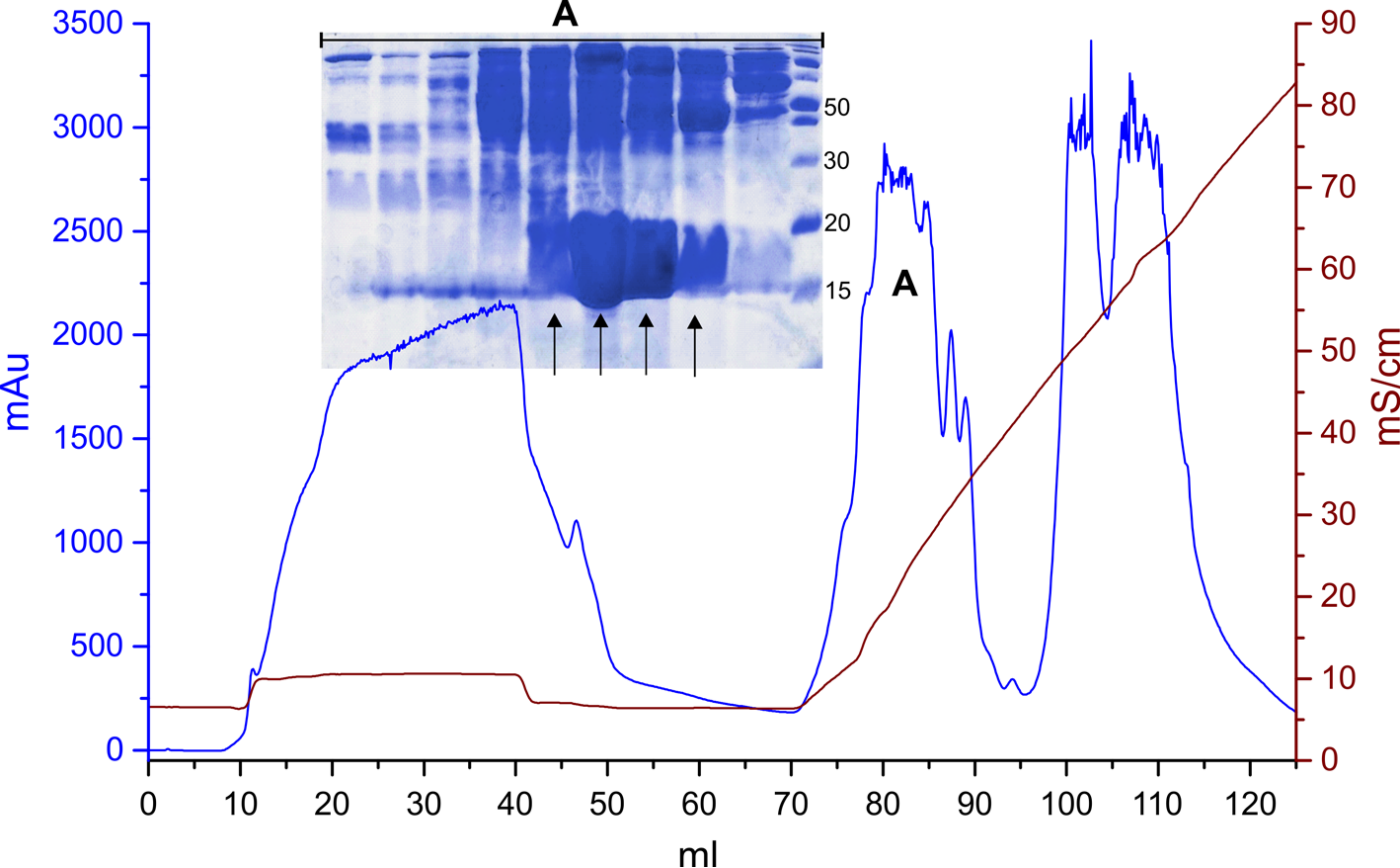
Initial step of lactoglobulin purification on *Fractogel TMAE* (*S*) (the example of sBlgB). Proteins were eluted using 0–50 % gradient of 2 M NaCl in 50 mM phosphate pH 6.5. sBlgB was eluted in peak A (fractions marked by *arrows* on SDS-PAGE gel)

The described below purification protocol was applied to rBlgB (product of pETDuet-1/*DsbC*/*BlgB*) and sBlgB (product of pETDuet-1/*DsbC*/L1A/I2S-*BlgB*). This purification protocol follows the procedure described by [12] with some modifications. Cell pellet (approximately 4–5 g) were re-suspended in 20 ml of 50 mM phosphate buffer (pH 6.5) containing PMSF. Cells were disrupted by sonication (3 × 5 min) and centrifuged at 15,000×*g*. Clarified cell lysate was loaded on chromatography XK 16/20 column (*GE Healthcare*) packed with *Fractogel EMD TMAE* (*S*) resin (*Merck Millipore*), equilibrated previously with the 50 mM phosphate buffer pH 6.5. Proteins were eluted using a linear gradient of 2 M NaCl (Fig. 1). Collected fractions were analysed by SDS-PAGE, and those containing β-lactoglobulin were pooled and dialysed overnight to 50 mM phosphate buffer pH 7.5.

**Fig. 2:**
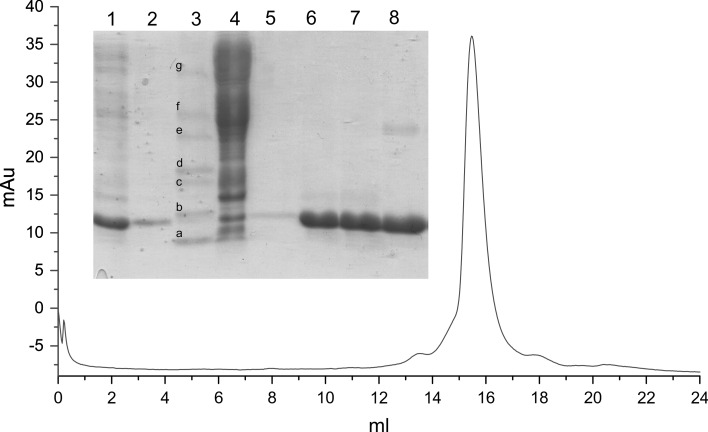
SDS-PAGE analysis of rBlgB purification: *Line 1* pooled fractions collected in anion-exchange chromatography, *line 2* supernatant after salting out by 7 % (w/v) NaCl, *line 3* marker (*a* 14.2 kDa, *b* 20.1 kDa, *c* 24 kDa, *d* 29 kDa, *e* 36 kDa, *f* 45 kDa, *g* 66 kDa), *line 4* pellet after salting out by 7 % (w/v) NaCl, *line 5* pellet after salting out by 30 % (w/v) NaCl re-suspended in phosphate buffer (diluted rBlgB), *line 6,* *7* concentrated rBlgB (15 and 20 mg/ml), *line 8* natural β-lactoglobulin purchased from Sigma-Aldrich (20 mg/ml). Size exclusion chromatography on *Superdex200 10/300 GL* performed for rBlgB#1 (corresponds to *lines* *6* and *7* on gel)

After dialysis, lactoglobulin concentration was estimated by measurement of A_280_ (*ε* = 17 600 M^−1^ cm^−1^ [17]). The protein solution was diluted to concentration ~1 mg/ml with phosphate buffer, and then, 0.1 M HCl was added dropwise with continuous stirring, to achieve a pH of 2.6. Solid NaCl was added to 7 % (w/v) to precipitate first protein fraction. Precipitated proteins were removed by centrifugation at 15,000×*g*, and the supernatant containing β-lactoglobulin was recovered. Solid NaCl was added to reach a concentration of 30 % (w/v), and proteins precipitated at this step (containing predominately rBlgB or sBlgB, Fig. 2) were harvested by centrifugation at 15,000×*g*. The pellet was re-suspended in a small volume of 50 mM phosphate buffer pH 7.5 and dialysed overnight at 4 °C. Protein purity and homogeneity were checked by SDS-PAGE and size exclusion chromatography at *Superdex75 10/300 GL* (*GE Healthcare*) (Fig. 2).

Chemical, N-terminal protein sequencing by the Edman method (*Procise 491 Sequencer, Applied Biosystems*, *BioCentrum*, Poland) of first five amino acids was performed for rBlgB being a product of pETDuet-1/*DsbC*/*BlgB* expression. Prior to sequencing, protein was deformylated using the protocol described by Hirano et al. [18] (Fig. S1).

The molecular weight of lactoglobulin (rBlgB and sBlgB) was determined using mass spectrometry (MS). Mass spectra were recorded with the use of an MALDI-TOF/TOF mass spectrometer (*ultrafleXtreme, Bruker Daltonics*) equipped with smartbeamTM laser operated at 1 kHz repetition rate. Spectra acquisition and pre-processing were performed with *flexControl* and *flexAnalysis* software, both from *Bruker*. Spectra were registered within *m*/*z* range of 5000–20,000 in a linear positive mode with 25 kV acceleration voltage. Multiple charge state analysis (MCSA) [19] was employed for the determination of protein mass weight. 2,5-Dihydroxyacetophenone (DHAP) was used as a matrix, while Protein Calibration Standard I (*Bruker*) was exploited for calibration of the TOF analyser.

### Purification Protocol #2

**Fig. 3:**
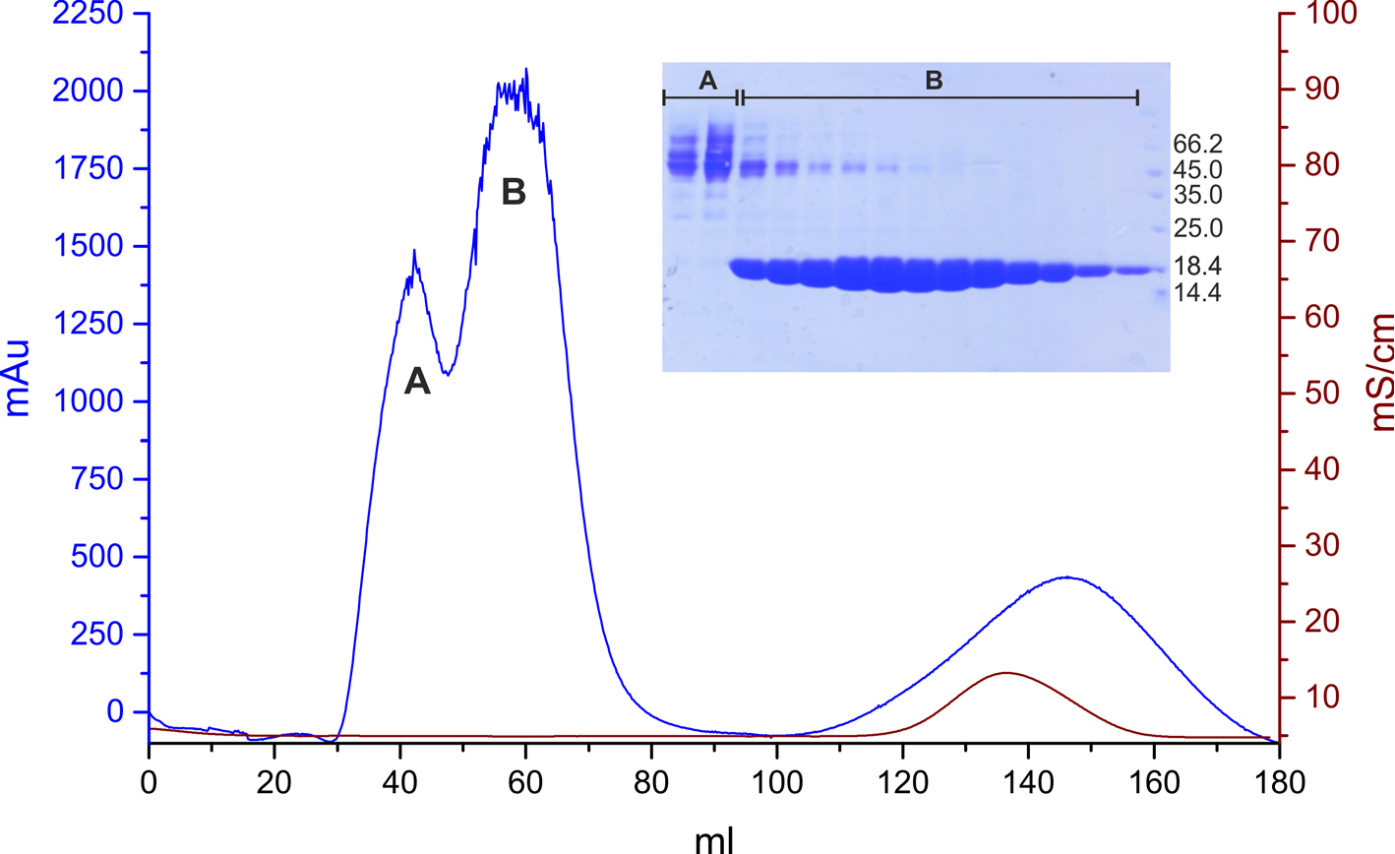
Purification of sBlgB#2 on *Sephadex G75*. Fractions *B* contained almost pure L1A/I2S-BlgB

This protocol was applied only to L1A/I2S-substituted variant (sBlgB). The initial steps of protein purification were the same as in protocol #1, but after ion-exchange chromatography at *Fractogel EMD TMAE* (*S*), instead of salting out by NaCl, the second chromatography step was applied. Fractions containing lactoglobulin, eluted from an anion-exchange resin, were analysed by SDS-PAGE, and those containing the highest content of L1A/I2S were pooled and loaded on XK 16/20 column (*GE Healthcare*) packed by *Sephadex G75* (*Pharmacia*). Proteins were eluted using 50 mM phosphate buffer pH 6.5 (Fig. 3). Eluted fractions were analysed by SDS-PAGE (insert in Fig. 3).

### Circular Dichroism

CD spectra of rBlgB and sBlgB were recorded at 20 °C on a JASCO J-710 spectropolarimeter using 50 mM phosphate buffer pH 6.5 or 7.5. In the case of pH-dependent measurements, buffer containing 20 mM acetate, 20 mM phosphate, 20 mM Tris and 20 mM borate was used and the pH was adjusted to the desired value by adding HCl or NaOH. Each sample was prepared at least a half-hour before measurement.

The protein concentrations 10–15 μM and the 200 µm path length were used for far-UV measurements, while the protein concentrations 50 μM and the 5 mm path length were used for near-UV measurements. Three scanning acquisitions were accumulated and averaged to yield the final spectrum. CD spectra were corrected for the buffer baseline. The ellipticity was converted to a difference in extinction coefficients. The spectra were normalised to the peptide bonds concentration for far-UV and the protein concentration for near-UV.

The secondary structure composition was estimated using CDPro spectra deconvolution software developed by Sreerama and Woody [20]. The best fits were obtained using CONTINLL algorithm and the SDP42 reference spectrums data set.

### Thermal Denaturation

Equilibrium thermal unfolding of the sBlgB#2 was monitored by CD on a *JASCO J-710* spectropolarimeter using 1-mm path length quartz thermostated cuvette sealed with a Teflon stopper to avoid evaporation during thermal unfolding. The measurements were taken at 50 mM phosphate buffer pH 6.5. The temperature of the cuvette in the sample beam was controlled by Julabo circulating water bath. A signal at 200 nm was recorded as a function of temperature over a range of 20–95 °C. The heating rate in the experiments was 1.0 °C per minute. The raw temperature scans were processed using a first-order Savitzky–Golay algorithm of *Origin* software (version 9.1.0) with a window of 20 points to determine the first-order derivatives and *T*_m_ values as a mean value of three independent runs.

### Chemical Denaturation

Urea-induced unfolding of the protein was monitored by CD on a JASCO J-710 spectropolarimeter using 1-mm path length quartz cuvette. A signal at 220 nm was recorded in 50 mM phosphate buffer pH 6.5 at room temperature. The samples were prepared 1 day before experiment by mixing a stock solution of the protein with 10 M urea solution in the same buffer to a final concentration of 5 µM sBlgB#2 and incubated overnight at room temperature. Global analysis of data in triplicates was conducted using *Origin* software assuming a two-state mechanism according to methods described in [21].

### Isothermal Titration Calorimetry

All ITC experiments, both for recombinant proteins and milk lactoglobulin used as a reference, were carried out at 25 °C using a VP-ITC instrument (MicroCal, Northampton, MA, USA). SDS binding experiments were performed according to methods described in our previous paper [22] using 50 mM phosphate buffer pH 6.5 or 50 mM Tris–HCl buffer pH 7.5. Data analysis was performed using *MicroCal Origin* scientific plotting software according to the model of the single set of identical independent sites. Standard deviations of determined parameters were calculated from at least two titration runs. In the case of data without typical sigmoidal dependence of heats versus reagents molar ratio, fixed value of enthalpy equal to −31.07 kJ/mol was used [22].

### Crystallization

Recombinant lactoglobulin was crystallised using vapour diffusion method in hanging drop set-up. Small (~0.1–0.2 mm), poorly diffracting crystals of rBlgB appeared only in drops formed by mixing 1–2 μl of protein (concentration between 12.5 and 25 mg/ml) and 1–2 μl of 1.8–3.0 M (NH_4_)_2_SO_4_ solution in 0.2 M Tris–HCl buffer pH from 7.8 to 8.8. Larger crystals (~0.4 mm) suitable for data collection were obtained in drops containing 1 μl of 1.34 M sodium citrate in 0.1 M Tris pH 7.5 and 1 μl of protein (30 mg/ml) for sBlgB#1 or 1 μl of 2.3 M (NH_4_)_2_SO_4_ in 0.5 M Tris–HCl pH 8.5 for sBlgB#2.

### Data Collection, Structure Solution and Refinement

X-ray diffraction data for rBlgB crystal were collected at MAX-LAB synchrotron facility at I911-3 beamline (1.00 Å). Crystals were washed in cryoprotectant (20 % v/v glycerol) and immediately transferred to nitrogen cryostream (100 K). Data were recorded on CCD MAR 165 detector and processed using XDS [23] and HKL2000 [24].

Diffraction data for sBlgB crystals were collected at *SuperNova* diffractometer (*Rigaku Oxford Diffraction*) using CuKα radiation (1.54 Å) generated by microfocus radiation source (0.8 mA and 50 kV). Data were collected at 120 K using 20 % v/v glycerol as a cryoprotectant and recorded on 135-mm Atlas CCD detector. Data were processed using *Crysalis*^*Pro*^ (*Rigaku Oxford Diffraction*) and *Scala* [25] form *CCP4* package [26].

Structures of rBlgB, sBlgB#1 and sBlgB#2 were solved by molecular replacement with *Phaser* [27] using bovine β-lactoglobulin structure 1BSY or 1B8E as a starting model. Structures were refined by *Refmac5* [28], while Fourier maps were investigated using *Coot* [29]. Statistics of data collection and structure refinement are summarised in Table 1. Structures were deposited in Protein Data Bank (PDB) as entries: 5K06, 5HTD, 5HTE.

**Table 1:** Statistic of data collection and structure refinement

	rBlgB	sBlgB#1	sBlgB#2
PDB	5K06	5HTD	5HTE
Endogenous ligand	Yes	Yes	No
Space group	P6_4_22	P3_2_21	C222_1_
Unit cell [Å]	*a* = 106.65	*a* = 53.49	*a* = 54.85
	*b* = 106.65	*b* = 53.49	*b* = 79.09
	*c* = 59.31	*c* = 111.04	*c* = 65.70
Resolution range [Å]	9.98–2.50 (2.54–2.50)	13.75–2.50 (2.62–2.50)	13.79–2.40 (2.49–2.40)
Completeness [%]	99.6 (100)	98.9 (98.8)	97.4 (97.8)
Redundancy	19.7 (20.4)	2.8 (2.3)	2.5 (1.8)
*R*_merge_	0.073 (0.228)	0.072 (0.348)	0.047 (0.616)
*I*/*σI*	64.7 (16.1)	8.4 (1.8)	13.7 (2.0)
Reflections (test set)	6058 (1043)	5367 (1293)	4456 (1187)
*R* [%]	21.3	20.1	21.1
*R*_free_ [%]	32.4	27.2	30.3
rmsd bonds [Å]	0.012	0.007	0.010
rmsd angles [°]	1.676	1.116	1.465
Ramachandran statistics			
Favoured [%]	93	95	90
Allowed [%]	4	5	10
Outliers [%]	2	0	0

## Results

### Expression in *Origami B* (*DE3*) and Purification of Recombinant β-Lactoglobulin

The optimal expression time maximising the amount of soluble protein produced in *Origami* cells was 3–4 h for rBlgB and 18–20 h (overnight) for sBlgB at room temperature. To monitor expression level, samples of cell culture were collected and analysed by SDS-PAGE. Inspection of gels (Fig. S2) clearly showed that bands having a molecular weight about 24 kDa (DsbC) and 18 kDa (BlgB) appeared 1 h after induction and their intensity increased with time.

The typical yield of soluble protein (purity ≤90 %, Fig. 3) obtained from 4 to 5 g wet cells produced in 1 L culture medium was about 15–20 mg of rBlgB or sBlgB (protocol #1) and about 30–40 mg of sBlgB (protocol #2).

### Folding of Recombinant Bovine β-Lactoglobulin Produced in *Origami B* (*DE3*)

**Fig. 4:**
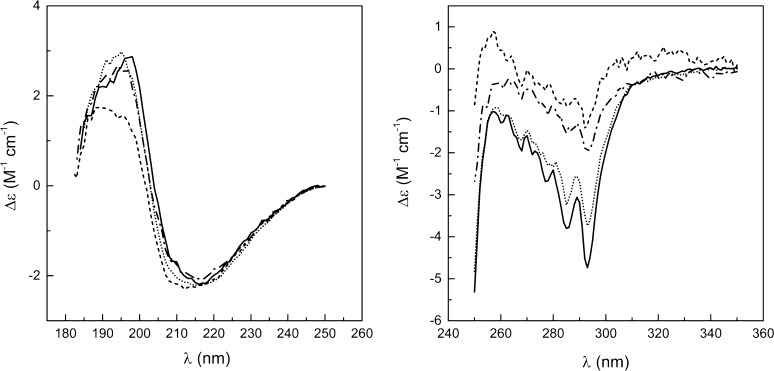
Far-UV (*left panel*) and near-UV (*right panel*) CD spectra of BlgB (*solid line*, from [27]), rBlgB (*dash-dot line*), sBlgB#1 (*dashed line*) and sBlgB#2 (*dotted line*) in 0.05 M phosphate buffer pH 7.5, 20 °C. Δ*ε* was normalised by peptide bonds or protein concentration

The correctness of recombinant lactoglobulin (rBlgB and sBlgB) folding was routinely verified by CD spectra in the far and near-UV range (Fig. 4). The position of extremes did not depend on the sample, while amplitudes strongly depended on the type of the protein and purification protocol indicating a more dynamic structure of all recombinant proteins in comparison with milk β-lactoglobulin. Despite these differences, the quantitative analysis of spectra using *CDPro* software gave for all analysed recombinant proteins contents of the secondary structures being in agreement with that calculated from crystallographic data (Table S1). The best compliance to signal observed for milk protein at the near-UV range was for sBlgB#2. The amplitude of both troughs at about 286 and 293 nm and their ratio are close to measured earlier [30, 31].

### Ligand Binding by Recombinant Bovine β-Lactoglobulin Produced in *E. coli*

**Fig. 5:**
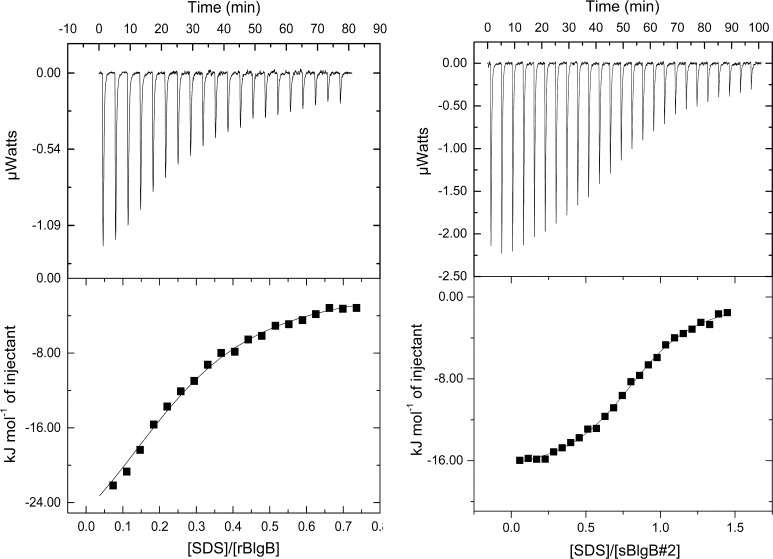
Calorimetric isotherms of SDS binding to rBlgB (*left panel*) and sBlgB#2 (*right panel*). The experiments were performed at 25 °C in 50 mM Tris–HCl buffer pH 7.5 and 50 mM phosphate buffer pH 6.5, respectively. The protein and ligand concentrations were 28 and 500 µM for rBlgB and 37 and 100 µM for sBlgB#2. The *solid line* represents the best fit of experimental data to the one site binding model. A fixed value of enthalpy (−31.07 kJ/mol) was used in case of rBlgB

Because SDS binding to lactoglobulin is well characterised in the literature [22, 32], to verify the ability of the recombinant protein (rBlgB, sBlgB#1 and sBlgB#2) to bind ligands, we selected it as a model ligand. Observed initial heat release (Fig. 5) was consistent with effect measured for BlgB isolated from milk [22, 32, 33]. The binding constant *K*_a_ and thermodynamic parameters of SDS binding are presented in Table 2. The stoichiometry for rBlgB and sBlgB#1 was in the range from 0.15 to 0.4, while for sBlgB purified by protocol #2 stoichiometry was above 0.8.

**Table 2:** Thermodynamic parameters of SDS binding

Ligand binding					
	*n*	*K*_a_ (×10^5^ M^−1^)	Δ*G* (kJ/mol)	Δ*H* (kJ/mol)	*T*Δ*S* (kJ/mol)
rBlgB^a^	0.17–0.41	5.3 ± 1.5	−32.6 ± 0.7	−31.07^c^	n.d.
sBlgB#1^a^	0.19–0.38	1.6 ± 0.7	−29.5 ± 0.9	−31.07^c^	n.d.
sBlgB#2^b^	0.82–0.84	4.2 ± 0.2	−32.1 ± 0.1	−17.6 ± 0.2	14.54 ± 0.1
Milk BlgB^b^	1.01–1.05	3.6 ± 0.6	−31.7 ± 0.5	−20.3 ± 0.1	11.45 ± 0.2

^a^50 mM Tris–HCl, pH 7.5

^b^50 mM phosphate, pH 6.5

^c^A constant value taken from [22]

### Crystal Structure of rBlgB Purified by Protocol #1

**Fig. 6:**
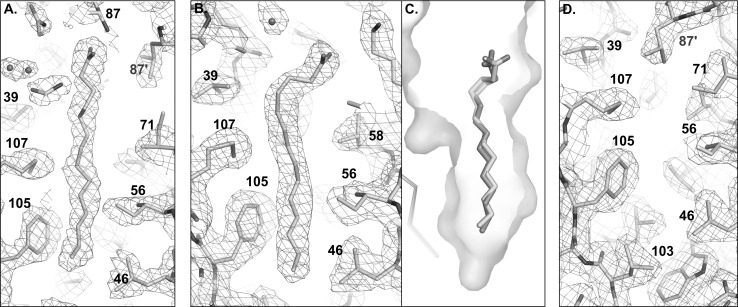
Electron density map around endogenous ligand bound in β-barrel of **a** rBlgB (1.2 σ) and **b** sBlgB#1 (1.0 σ). **c** The position of ligand in sBlgB#1 (*cyan*) and in milk lactoglobulin complex with myristic acid (*orange*, PDB: 3UEV). **d** Electron density map (1.0 σ) around residues in empty β-barrel in sBlgB#2 (Color figure online)

Crystals of rBlgB obtained from ammonium sulphate poorly diffract X-rays, and data collection required the use of synchrotron radiation. The unit cell symmetry P6_4_22 was not observed previously for β-lactoglobulin. The asymmetric unit contains one protein chain (Fig. 6). With the exception of GH loop which is disordered, the electron density is well defined for entire protein chain and shows that disulphide bridges are correctly formed. The density also allowed to resolve two alternative conformations of N-terminal part of the polypeptide chain located on crystallographic twofold axis. Additional electron density, not observed previously in structures of bovine milk lactoglobulin, was found near N-terminal fragment of protein molecules. Mass spectrometry revealed that molecular weight [M + H]^+^ of rBlgB was 18,409.8 (±5) Da, about 128.6 Da more than calculated from its sequence using *Compute pI/Mw* from *Expasy* server. This difference corresponds within experimental error to the mass of methionine (132 Da). Chemical sequencing (see “Purification Protocol #1” section and Fig. S1) confirmed the presence of the uncleaved N-terminal methionine Met0. Fourier maps also showed unexpected elongated density in the hydrophobic pocket of β-barrel. 10–16-carbon long fatty acids were modelled, and the best fit has been observed for 14-carbon myristic acid (MYR) (Fig. 6). As the rBlgB was crystallised without the addition of potential ligands, it must have an endogenic origin.

### Crystal Structure of sBlgB Purified by Protocol #1

The space group P3_2_21 and unit cell parameters of sBlgB crystals correspond very well to the trigonal form of natural bovine β-lactoglobulin. The crystal structure was in agreement with MS results, indicating that N-terminal methionine has been successfully cleaved (determined m.w. was 18,211.7 ± 5 Da, while the calculated was 18,213.05 Da). The electron density is well visible for almost entire protein chain; the exception is flexible loop GH. Electron density maps confirmed that disulphide bridges, between residues Cys66–Cys160 and Cys106–Cys119, are correctly formed, so the applied system of BlgB co-expression with DsbC works efficiently for lactoglobulin sequence carrying mutation L1A/I2S.

Similarly to previous structure, electron density maps revealed the presence of a ligand in the β-barrel, even though no ligand was added to protein solution and crystallization drops (Fig. 6). The electron density in the binding pocket was similar to that observed in the structures of lactoglobulin–fatty acid complexes, and it has been interpreted as a myristic acid [22, 34]. Superposition of sBlgB structure with BlgB-MYR complex (PDB: 3UEV) revealed almost the same position of ligand in both structures (Fig. 6c).

### Crystal Structure and Properties of sBlgB Purified by Protocol #2

**Fig. 7:**
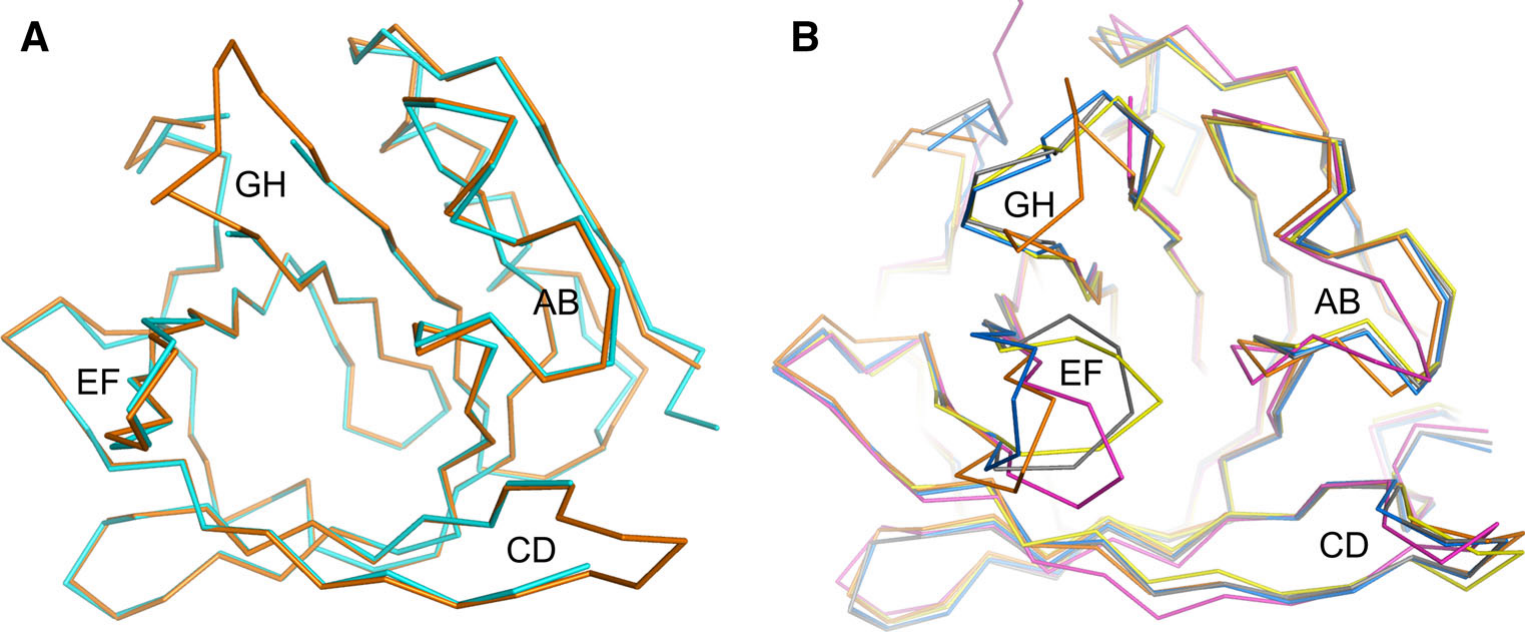
**a** Superposition of sBlgB#2 structure (*cyan*, C222_1_) and natural lactoglobulin (*orange*, PDB: 1B8E, C222_1_). **b** Superposition of lactoglobulin structures: rBlgB (chain C: *magenta*), PDB: 1BSY (*blue*), PDB: 3BLG (*grey*), PDB: 1B8E (*orange*), PDB: 1BEB (*yellow*) (Color figure online)

The crystals obtained using recombinant sBlgB purified by protocol #2 had space group symmetry C222_1_, the same as observed several times for natural, unliganded protein [35, 36]. The electron density is well defined except for flexible loops CD and GH and disordered C-terminal fragment 155–162. Contrary to protein purified by protocol #1, there is no evidence of any ligand bound in the binding site (Fig. 6d). Superposition of sBlgB#2 and three natural β-lactoglobulin structures of the same space group symmetry gave r.m.s.d. values for *C*_α_ in the range 0.39–0.47 Å (Fig. 7a, b ).

ITC experiments performed using SDS as a model ligand provided binding parameters (Table 2) being in good agreement with parameters obtained by us in reference experiments with milk Blg in the same conditions and others [33].

To further characterise sBlgB#2 protein, we also performed thermal and chemical denaturation experiments. The results of urea-induced denaturation presented in Table 3 show that the stability of a recombinant protein is lower than of milk protein by about 11.5 kJ/mol, but their *C*_mid_ values are similar. Also, the thermal denaturation of the protein analysed as the temperature dependence of the CD signal derivative measured at 200 nm indicates lower stability of sBlgB#2 in comparison with milk protein. The *T*_m_ value decreased from 79.4 ± 1.4 °C for milk protein to 72.8 ± 0.4 °C for sBlgB#2. A storage stability of sBlgB#2 was monitored by measurements of CD spectra. We did not observe any change in a term of 2 weeks at room temperature and at 4 °C (Fig. S3).

**Table 3:** Thermodynamic parameters of protein chemical unfolding for protein concentration in range from 0.1 to 0.73 mg/ml

Experimental conditions		Chemical denaturation parameters			
pH	Ionic strength (mM)	Δ*G*_D_ (kJ/mol)	*m* (kJ/mol M)	*C*_mid_ (M)	Ref.
*Recombinant sBlgB#2*					
6.5	50	16.2 ± 2.4	3.9 ± 0.6	4.18	This work
*Natural (milk) BlgB*					
6.5	50	27.7 ± 5.4	6.5 ± 1.3	4.26	This work
7.0	50	25.0 ± 1.5	6.5 ± 0.35	3.8	[47]
6.2	10	12.1 ± 5.5	3.77 ± 1.26	3.2 ± 0.8	[21]
6.85	10	27.8	6.4	4.35	[48]
8.3	10	20.5 ± 4.6	5.02 ± 0.85	3.9 ± 0.4	[21]

Because it is known that pH strongly modifies the Blg properties, especially its ability to bind ligands [37–39], CD signal versus pH was measured for sBlgB#2 and natural BlgB isolated from milk and no significant differences have been found (Fig. S4).

## Discussion

### Expression of β-Lactoglobulin in *E. coli*

Although recombinant bovine lactoglobulin has been successfully produced in eukaryotic expression systems (*Pichia pastoris*) [7], the production of correctly folded protein in bacteria was unsuccessful until 2010. The breakthrough was made by Ponniah et al. [12] who found that BlgB must be co-expressed in *E. coli Origami* cells together with bacterial disulphide bond isomerase (DsbC). We used this expression system and purification protocol without any major modifications to produce recombinant lactoglobulin in our laboratory. However, we have noticed that obtained protein (rBlgB) had properties different than protein isolated from milk.

The ITC experiments gave *K*_a_ values of SDS binding to rBlgB in agreement with values obtained using milk protein in the same conditions [22, 33], but the binding stoichiometry in the range 0.17–0.41 was much lower than expected. This result could be explained by partially incorrect folding of the protein or by the occupation of the binding pocket by endogenous ligand. The second hypothesis has been confirmed by the crystal structure of rBlgB which showed the presence of endogenous aliphatic ligand bound inside the β-barrel (Fig. 6a).

Mass spectrometry revealed that molecular weight [M + H]^+^of rBlgB was higher than the expected value by 128.59 Da indicating the possible presence of uncleaved N-terminal methionine. The chemical sequencing of rBlgB unambiguously confirmed this hypothesis (Fig. S1). The uncleaved N-terminal Met is sometimes observed in recombinant proteins, and its impact on protein properties and stability can be positive, insignificant or negative. Uncleaved N-terminal methionine was found e.g. in recombinant hen egg white lysozyme produced in *E. coli.* In this case, it was found that protein with additional Met had lower refolding rate and solubility in comparison with wild-type protein [40]. The N-terminal methionine present in recombinant rubredoxin from *Pyrococcus furiosus* had a moderate effect on protein thermal stability and structure [41]. Crystal structure of recombinant rubredoxin showed that extra methionine has changed only the hydrogen bond pattern in its close neighbourhood [41].

The positive effect of the N-terminal Met on protein stability was observed in recombinant ribonuclease A (RNase A) [42]. Enzyme containing uncleaved N-terminal Met had enzymatic activity close to that of bovine RNase A, while its transition midpoint for thermal unfolding was 1.5 °C higher than that of natural bovine RNase A [42]. Contrary, N-terminal methionine, which was detected in recombinant goat α-lactalbumin expressed in *Escherichia coli*, remarkably decreased the stability of the protein and increased its apparent net negative charge. It also affected the packing of α-lactalbumin molecules in the crystalline phase and altered crystal symmetry [43].

Likewise, in rBlgB uncleaved starting methionine negatively influenced properties of protein, affecting not only binding stoichiometry but also crystallization conditions and intermolecular interactions in the crystalline phase. The crystallization trials showed that rBlgB could be crystallised only from the high concentration of ammonium sulphate (2.8–3.0 M) and such conditions produced small, poorly diffracting crystals with morphology and symmetry substantially different than observed for natural protein.

The origins of such differences have become understood later, after careful analysis of crystal structure. They are the effect of partial disorder around N-terminus fragments with uncleaved Met, which also affects positions of other residues. Superposition of rBlgB and known structures of milk lactoglobulin (1BSY, 1BEB and 3NQ3) revealed that N-terminal parts of the protein chain and AB loop have position shifted by about 2–5 Å in comparison with its location in trigonal structures.

Hirel et al. [44] have found that in bacteria cells, the catalytic efficiency of N-terminal methionine excision by MAP (l-methionine amino peptidase) depends on the nature of the second amino acid in the polypeptide chain. Efficiency of MAP decreases when the side chain length of the penultimate amino acid increases. This dependence is related to the structure of the enzyme active site in which each subsite is able to accommodate side chain with specified dimensions [44, 45]. The rate of N-terminal Met processing is the highest for residues possessing short side chains (Gly, Ala, Pro, Ser, Thr, Val) while the sequence of BlgB starts with relatively large leucine and isoleucine. To eliminate N-terminal Met, we have engineered two mutations (L1A/I2S), which were designed according to more recent findings by Frottin et al. [45]. They observed that most efficient cleavage of N-terminal Met occurs when an N-terminal sequence is Met–X–Y, where X is Gly, Ala, Pro, Ser and Y is Gly, Ala, Trp, Met or Ser. We have decided to replace Leu1 by Ala, which is the nonpolar residue with shorter side chain, so such substitution in contrast to Gly, Pro or Ser should not affect the protein properties significantly. In the second position, Ile has been replaced by Ser, to avoid residue with large side chain (Trp, Met) and duplication of residues (Ala).

The presence of additional Met at N-terminus was not reported by Ponniah et al. [12] who checked only CD and NMR spectra of recombinant lactoglobulin. These techniques are also not sensitive enough to show the differences, especially when the core of protein made of β-barrel is correctly folded. Interestingly, the presence of uncleaved Met was also not recognised by Crowther et al. [46] who expressed recombinant caprine β-lactoglobulin using the same DsbC-co-expression system. The recombinant goat protein was crystallised, and structure was solved with ultra-high resolution (PDB: 4TLJ). But careful investigation of electron density maps that are available for this structure on *Electron Density Server* revealed an extra electron density near N-terminus in which methionine can ideally be fitted, as predicted.

### Purification Protocols of Recombinant β-Lactoglobulin

The new lactoglobulin variant carrying substitutions L1A/I2S at N-terminus (sBlgB) has been produced and purified by two different protocols. The sBlgB purified by protocol #1, based on the method proposed by Ponniah et al. [12], had spectroscopic properties corresponding very well to milk protein (Fig. 4) and crystallised in the conditions typical for natural protein. However, ITC tests were constantly showing a very low stoichiometry of SDS binding, with the values similar to the observed for rBlgB (Table 2). These results indicated that the binding site of sBlgB#1 was probably occupied by endogenous ligand. This hypothesis was verified by the crystal structure, which confirmed the presence of the endogenous ligand in the β-barrel. Elongated electron density observed in the binding site was interpreted as 14-carbon myristic acid. However, its origin is unknown. It might be either fatty acid present in cytoplasm and bound during expression or fatty acid from disrupted cell membranes which has bound during purification. The second possibility seems to be the most probable because the modification of a purification protocol allowed to obtain the sBlgB with empty β-barrel (Fig. 6d).

Purification of L1A/I2S BlgB variant by protocol #1 requires one chromatography step and two time-consuming overnight dialysis procedures, but the efficiency of purification is relatively low. For these reasons, we have decided to modify this protocol. In the protocol #2, instead of two salting out and dialysis steps, we have used the size exclusion chromatography on Sephadex G75 at pH 6.5. Such replacement allowed us to obtain the recombinant L1A/I2S BlgB variant with the unblocked binding site (sBlgB#2). The protocol #2 also eliminates the need of strong acidification of protein solution to pH 2.6 which was necessary to precipitate *E. coli* proteins (Fig. 2). Instead, in protocol #2, bacterial proteins were separated from lactoglobulin in gel filtration step, because most of them have molecular masses higher than lactoglobulin (Fig. 3).

The observation that protocol #2 allows obtaining a protein with empty β-barrel indicates that ligand was trapped in the BlgB β-barrel in the purification process rather than during expression in *E. coli*. Binding of fatty acid might occur e.g. at cell lysis step or later, at low-pH salting out. Another possibility is that in the acidic environment most of the lactoglobulin molecules, especially those that were not stabilised by a fatty acid, have been denaturated. Only a small fraction of lactoglobulin molecules with a bound ligand was stable enough to remain soluble. Such hypothesis would also explain low efficiency of lactoglobulin purification by protocol #1.

### Binding Properties and Stability of sBlgB#2

As ITC showed it, sBlgB#2 can bind model ligand (SDS) with the stoichiometry 0.8 (Table 2). The value of the stoichiometry lower than 1.0 is in agreement with our previous data [22, 34] and was also observed in other experiments [29]. The binding constant *K*_a_ is in good agreement with reference data obtained from milk protein [22, 33]. The values of the enthalpy and entropy changes determined for sBlgB#2 are higher by about 3 kJ/mol but compensate, giving Δ*G* almost the same as observed for milk protein (Table 2). These data show that recombinant protein, despite substitutions L1A/I2S introduced at N-terminus, has binding properties comparable to natural β-lactoglobulin isolated from milk.

**Fig. 8:**
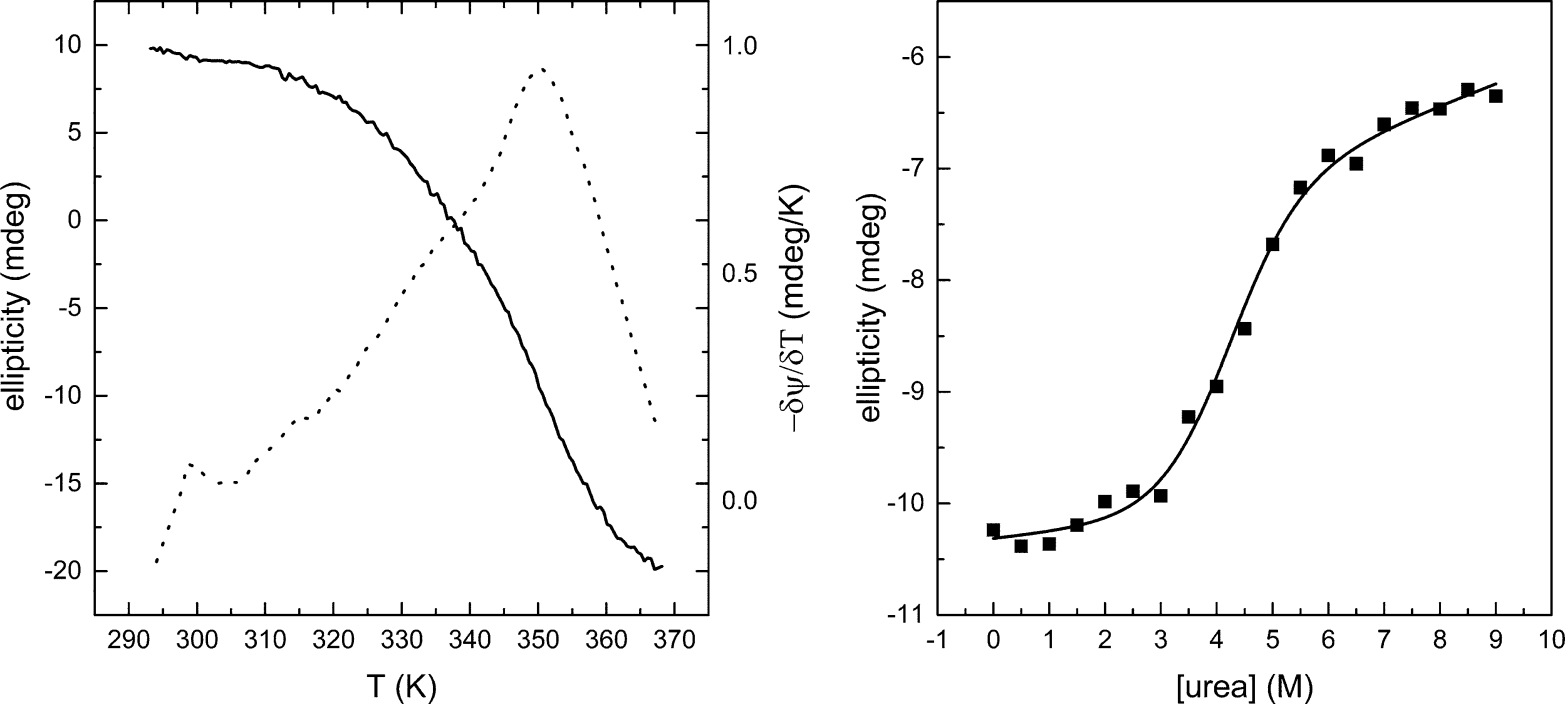
Unfolding of sBlgB#2 studied by circular dichroism spectroscopy. The experiments were performed at 20 °C in phosphate buffer at concentration 0.05 M and pH 6.5. *Left* *panel* the thermal unfolding of 12 µM protein reported at 200 nm (*solid line*) and its derivative (*dotted line*). *Right panel* urea concentration induced unfolding of 10 µM protein reported at 220 nm. The *solid line* represents the best fit of the data to the two-state model of denaturation

The chemical stability of sBlgB#2 determined by urea-induced denaturation (Fig. 8) can be described by the two-state model and is lower than measured by us for commercially available milk protein (Table 3). Survey of results reported by other authors [21, 47, 48] shows considerable discrepancies, both negative and positive, in the *C*_mid_ values even for data determined spectroscopically at similar experimental conditions and analysed with the use of the same model of denaturation (Table 3). It indicates that not only experimental conditions but also protein origin might influence obtained values, and such finding suggests that lower than expected value of chemical stability of sBlgB#2 cannot be interpreted as the negative attribute of recombinant protein.

Similar ambiguous observation concerns lactoglobulin thermal denaturation parameters (Fig. 8). Determined *T*_m_ value has pointed lower thermal stability of sBlgB#2 in comparison with milk protein. On the other hand, the thermal stability of milk lactoglobulin determined previously spectroscopically at similar experimental conditions varies in the range 68–80.1 °C [49].

Our studies also show that the CD signal dependence on pH for sBlgB#2 was in agreement with the one recorded for milk protein (Fig S4). We have limited our investigations to one method and pH range from 4 to 10, in which only two conformational transitions of lactoglobulin could be analysed: so-called N-to-Q transition [50] and Tanford transition [37]. High similarity of signals from both proteins and their consistency with data measured earlier [38] indicate in sBlgB#2 and milk protein the same nature of the conformational changes.

## Conclusions

High similarity of L1A/I2S BlgB purified by protocol #2 to natural protein has been confirmed by CD spectra, calorimetric studies, crystallization conditions and crystal structure. Because thermodynamic parameters of ligand binding are sensitive to pH, ionic strength and temperature, both sBlgB and milk protein used as a reference were investigated in the same conditions. All results confirmed that substitutions of N-terminal residues did not influence stability and binding properties of the protein significantly.

Therefore, it can be concluded that sBlgB#2 is a good substitute for natural lactoglobulin which can be used in the biochemical, biophysical, food and nanomaterials studies. It is also an excellent starting model for further engineering which would change its binding affinity towards selected ligands. New, engineered lactoglobulin with mutations in region of the binding pocket can potentially have an application in medicine.

## Electronic supplementary material

Below is the link to the electronic supplementary material.

Supplementary material 1 (DOCX 776 kb)

## Abbreviations

BlgB: Natural bovine β-lactoglobulin isoform B
rBlgB: Recombinant β-lactoglobulin isoform B
sBlgB: Recombinant β-lactoglobulin isoform B with N-terminal L1A/I2S substitutions
sBlgB#1: sBlgB purified by protocol #1
sBlgB#2: sBlgB purified by protocol #2
pETDuet-1/*DsbC*/*BlgB*: pETDuet-1 carrying rBlgB sequence
pETDuet-1/*DsbC*/L1A/I2S-*BlgB*: pETDuet-1 carrying sBlgB sequence
