# Supplementary material for: Engineered β-Lactoglobulin Produced in E. coli: Purification, Biophysical and Structural Characterisation
Source: Mol Biotechnol. 2016 Jul 5;58(10):605–18. doi: 10.1007/s12033-016-9960-z (PMC5035327; doi:10.1007/s12033-016-9960-z)
Supplement: Supplementary file 1 — Supplementary material 1 (DOCX 776 kb) [file 12033_2016_9960_MOESM1_ESM.docx]

**Supplementary data**

**Fig. S1.** Chemical sequencing of rBlgB (first five residues).


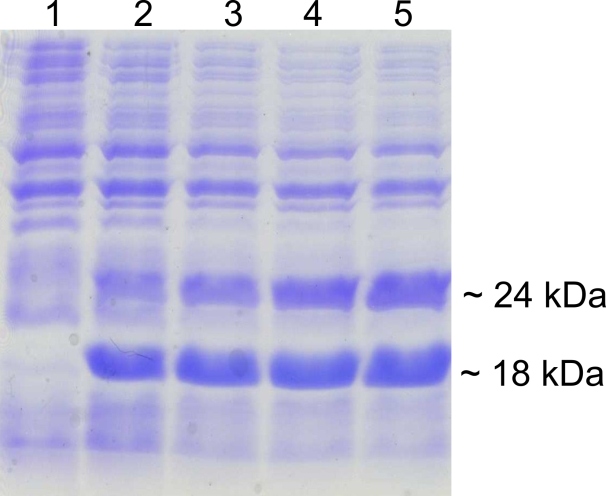


**Fig. S2.** Expression of recombinant lactoglobulin in *Origami* cells (an example of rBlgB). SDS-PAGE gel showing expression progress: line 1 - cell lysate before induction, lines - 2, 3, 4 and 5 cell lysate after 1h, 2h, 3h and 4h after IPTG addition.


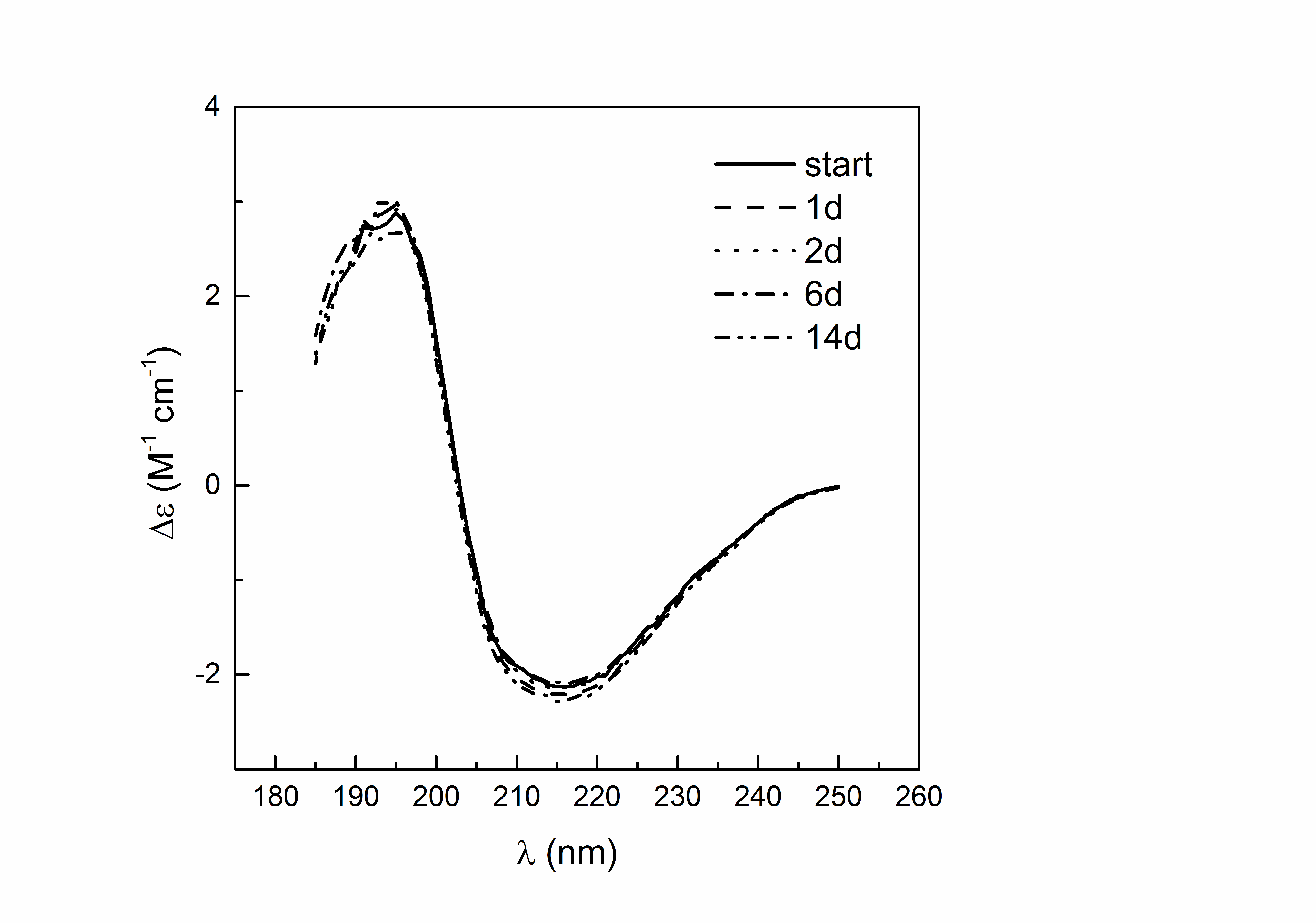

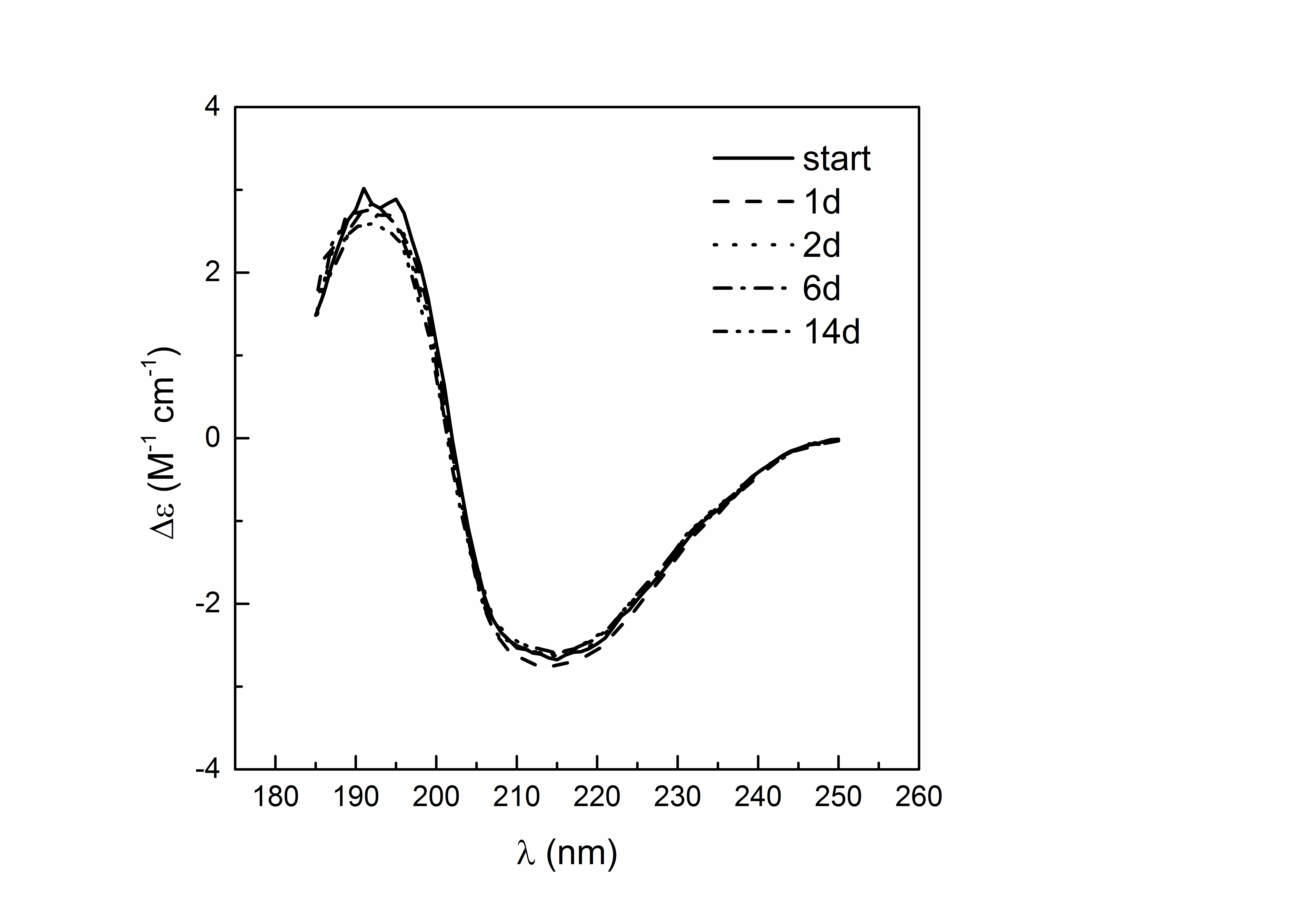


**Fig. S3.** Storage stability of sBlgB#2. CD spectra measured on consecutive days for samples stored at room temperature (left panel) or 4°C (right panel).

**Fig. S4.** pH dependence for sBlgB#2 (black symbols) and milk BlgB (open symbols) of the ellipticity at 200 nm. The experiments were performed at 20°C in buffer containing 20 mM acetate, 20 mM phosphate, 20 mM Tris and 20 mM borate.

**Table S1.** The secondary structures compositions of the recombinant lactoglobulin according to CDPro analysis of CD spectra.

|  | Helix | Sheet | Turn | Unregular |
| --- | --- | --- | --- | --- |
| rBlgB | 15% | 35% | 21% | 29% |
| sBlgB#1 | 13% | 38% | 24% | 25% |
| sBlgB#2 | 20% | 29% | 23% | 28% |
